# Liquid crystal-based structural color actuators

**DOI:** 10.1038/s41377-022-00937-y

**Published:** 2022-08-05

**Authors:** Pei Zhang, Laurens T. de Haan, Michael G. Debije, Albert P. H. J. Schenning

**Affiliations:** 1 Stimuli-responsive Functional Materials and Devices, Department of Chemical Engineering and Chemistry, Eindhoven University of Technology, P.O. Box 513, 5600 MB Eindhoven, The Netherlands; 2 Institute for Complex Molecular Systems, Eindhoven University of Technology, Den Dolech 2, 5600 MB Eindhoven, The Netherlands; 3 SCNU-TUE Joint Lab of Device Integrated Responsive Materials (DIRM), National Center for International Research on Green Optoelectronics, South China Normal University, Guangzhou, 510006 China

**Keywords:** Liquid crystals, Photonic crystals

## Abstract

Animals can modify their body shape and/or color for protection, camouflage and communication. This adaptability has inspired fabrication of actuators with structural color changes to endow soft robots with additional functionalities. Using liquid crystal-based materials for actuators with structural color changes is a promising approach. In this review, we discuss the current state of liquid crystal-based actuators with structural color changes and the potential applications of these structural color actuators in soft robotic devices.

## Abstract (web summary)

Liquid crystal-based actuators with structural color changes and the potential applications of these structural color actuators in soft robotic devices are reviewed.

## Graphical abstract

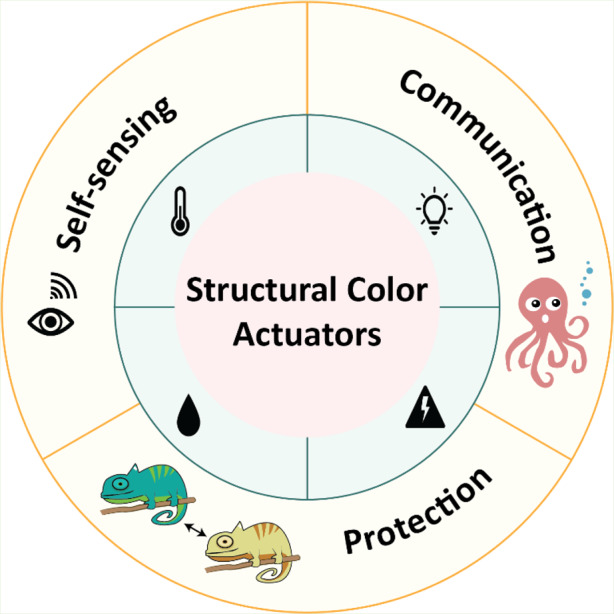

## Introduction

Camouflage is an important survival technique used by many animals in the wild. To this end, some animals have evolved the capability to change their overall appearance (color and shape) for signaling or surviving in challenging environments. For example, cephalopods are able to adapt both their body shape and color for camouflage: the mimic octopus can rearrange the form of its entire body to appear as another species, such as a flatfish or banded sea snake, in order to deceive predators^1^. Some animals, such as chameleons, can actively change their color for camouflage^2^. These features have inspired the development of soft actuators with tunable colors, as the capability to modify color can add more interactivity and feedback behavior, allowing disguise, self-sensing and communication.

**Fig. 1:**
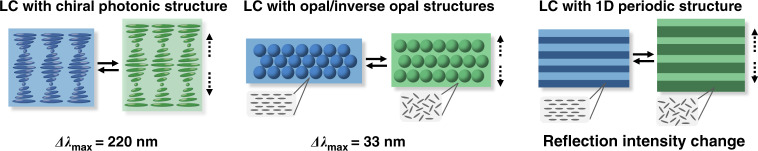
Overview of photonic LC materials for making structural color actuators and the corresponding mechanisms of actuation and structural color change. Left: LC with chiral photonic structure, including CLC, CNC and BPLC. The pitch/lattice spacing of photonic LCs increases when exposed to stimuli, causing dimensional and structural color changes. Middle: LC with opal/inverse opal structures; the spheres represent nanoparticles or air voids. Right: LC with alternating layers of two media with distinct refractive indexes. The disorder change of the LC when exposed to stimuli causes anisotropic deformation, leading to lattice spacing and structural color changes. *Δ****λ***_max_ represents the maximum structural color shift achieved in the reported literature

The two main sources of color are chemical, originating from wavelength-selective absorption or emission of light, and structural, deriving from physical interactions between light and periodic nanostructures. Both types of colors are widespread in organisms for camouflage, communication and reproduction. Actuators displaying chemical color change, commonly achieved by incorporating stimuli-responsive dyes^3–6^ and structural color actuators primarily using non-liquid crystal-based materials have both been recently reviewed^7^. In this work, we focus on structural color actuators based on liquid crystals (LCs), a promising class of stimuli responsive materials^8,9^. We highlight recent advances in structural color actuators using chiral photonic LCs, including cholesteric LCs (CLCs), cellulose nanocrystals (CNCs) and blue phase LCs (BPLCs), nematic LCs with opal/inverse opal structures, and nematic LCs with other microstructures (Fig. 1). Structural color and shape changes generated via manually applied mechanical forces will not be discussed in this review^10–24^. We introduce the basics of LCs, LC based soft actuators, and LC based structural color before discussing structural color actuators. Finally, we will discuss the challenges of existing systems and the future direction of structural color actuators for soft robotics.

## Basic concepts

### Liquid crystals

LCs can be considered as a state of matter between the liquid and solid phases, as they possess both the fluidic properties of a liquid and the order of crystals. LCs can be categorized into thermotropic, where the LC phase behavior dependents on temperature, and lyotropic, where the phase behavior depends on their concentration in solution. LC molecules can have different shapes; disc-like (discotic), rod-like (calamitic), lath-like (sanidic), and bent-core^25,26^.

Thermotropic LCs can exhibit different mesophases, including nematic, chiral nematic (cholesteric), and smectic (Sm) phases. Nematic phases exhibit only directional order of the molecules but no positional order: all the molecules tend to orient in one direction, referred to as the molecular director, ***n***. The nematic phase can be further organized in a cholesteric phase where planes of molecules exhibit a helical superstructure^25^. The lyotropic phase can exhibit similar mesophases as found for thermotropic liquid crystals: at low concentrations the monomers form an isotropic phase, while above a critical concentration, an LC phase is observed. Self-assembly of LCs can be controlled using a surface alignment layer, shear forces, stretching and electric or magnetic fields^8^. The alignment can be subsequently fixed by (photo)polymerization if the LC monomers contain polymerizable groups. Depending on the glass transition temperature (T_g_), LC elastomers (LCEs, T_g_ typically below room temperature) or LC networks (LCNs, T_g_ typically above room temperature) are obtained^27^.

### Liquid crystal-based soft actuators

Soft actuators are shape-changing responsive materials constructed from LC polymers that can be triggered by external stimuli to generate motions and forces^28^, and can operate both in air and underwater. Anisotropic shape deformation results from the disruption of the order of LC moieties^8^, leading to contraction parallel and expansion perpendicular to the molecular director. In some cases, the polymer may even undergo a phase transition to the isotropic state, leading to even greater shape changes. Molecular orientation can be programmed into the polymer to allow different actuation motions, including in-plane contraction, bending, and twisting, when triggered with different stimuli, including temperature, humidity or light^9^. Additionally, LC polymers can be designed to achieve re-programmability by introducing dynamic covalent adaptable networks^29,30^.

### Liquid crystal-based structural colors

Structural color arises from the interference of light with micro/nanostructures^31–35^. Structural color LC materials have been fabricated using CLCs, BPLCs, opal/inverse opal LC films, and LC polymers with 1D periodic multilayers, among others^36,37^. Thermotropic CLCs are commonly formed by adding a chiral dopant to nematic LCs, while lyotropic CNCs spontaneously form cholesteric phases^38,39^. In the cholesteric phase, the molecular director rotates periodically around an axis, forming a helical structure^40^. This helical structure can be either left- or right-handed, determined by the nature of the chiral dopant. The pitch (*P*) is defined as the unit length of one complete rotation (360°) of the director. The periodicity of the director rotation acts as a photonic micro/nanostructure and causes the material to reflect light of wavelength governed by:

1 $$\lambda = n \times P \times \cos \theta$$

where *λ* is the reflection wavelength, *n* is the average refractive index, *P* is the pitch and *θ* is the angle of incident light. CLCs show a degree of angular dependence with respect to the incident light, with a blue shift on deviating from the normal incident angle^41^. The spectral position of the reflection wavelength, that is, the structural color, can be controlled by adjusting the pitch. In CLC mixtures doped with chiral dopants, the pitch is determined by:

2 $$P = \frac{1}{{\left[ c \right] \times HTP}}$$

where [*c*] is the concentration and *HTP* is the helical twisting power of the chiral dopant. The *HTP* of a chiral dopant is indicative of its efficiency in inducing a twist in a nematic LC host. In low molar mass CLC mixtures, the pitch, and thus the resultant structural color, can be tuned with heat, electric fields, or light. Due to their fluidic nature, low molar mass CLCs are normally confined between two glass plates^41–43^ where further deformation is not possible. By dispersing CLC droplets in a polymer matrix^44^, a deformable freestanding film can be obtained. CLC mixtures using reactive monomers can also be polymerized to form polymer networks, resulting in freestanding films that retain their cholesteric phase.

Cellulose nanocrystals (CNCs), produced from plants and wood, are environmentally friendly, biocompatible materials. CNCs are crystalline nanorods with high aspect ratios capable of self-assembling in aqueous solution into a lyotropic CLC phase with left-handed helical twist due to the chiral 1,4-D-glucose units^38,39,45^, giving rise to the attractive optical properties of the cholesteric phase. However, CNC films are brittle and susceptible to redissolution in water. The solutions for these issues include incorporating CNCs with materials^23^ such as hydrogels or poly(ethylene glycol) to make flexible films, or to use the CNC as a chiral template^46–48^ to prepare materials where the cholesteric structure is preserved. When the CNCs are integrated with an elastomer, stretching-induced blue shift of the structural color can also be observed due to helical pitch compression^23,24^. In addition, the abundant hydroxyl groups in CNCs or hygroscopicity of the mesoporous polymer matrix allow the film to swell in water or water vapor.

In the cholesteric phase, the twisting of the helical structures is along the helical axis, with no twisting perpendicular to this axis. If the LC molecules can be twisted in both directions, double-twisted cylinders (DTCs) can be formed with defects, or disclinations between the cylinders in the LCs, known as BPs^49–52^. BPLCs are considered to be 3D photonic crystals and exhibit a selective reflection wavelength (*λ*) determined by^51,53^:

3 $$\lambda = \frac{{2na}}{{\sqrt {h^2 + k^2 + l^2} }}$$

where *n* is the average refractive index, *a* is the lattice constant of the BPLCs, and *h*, *k*, and *l* are the Miller indices of crystal orientation planes.

Opal structures with close-packed nanoparticles are another photonic construct that can display structural color^32^. Inverse opal films can be fabricated using close-packed nanoparticles such as silica as templates, and then removing the nanoparticles to create highly ordered voids which replicate the periodically ordered structure of the template and the photonic properties^54^. The reflection wavelength (*λ*) of the opals or inverse opals can be determined by^55,56^:

4 $$\lambda = \frac{{2d}}{m}(n_{{{{\mathrm{eff}}}}}^2 - \sin ^2\theta )^{1/2}$$

where *λ* is the position of the reflection wavelength, *d* is the distance between particle planes, *m* is the order of diffraction, *θ* is the angle of incident light, and *n*_eff_ is the mean effective refractive index.

Finally, a Bragg reflector consists of alternating high and low refractive index layers. This reflector displays structural color with the reflection wavelength (*λ*) determined by Eq. (4) ^57^, where *d* = *d*_h_ + *d*_l_, *d*_h_ and *d*_l_ are the thicknesses of the layers with high and low refractive indices, respectively.

The structural color of all these materials can be altered with temperature, humidity, light or mechanical deformations. In most cases, shape change of LC based structural colored materials automatically leads to structural color changes. In case of expansion, a red shift (longer wavelengths) of the reflection band is observed while shrinkage leads to a blue shift (see Eqs. (1), (3) and (4)).

### Liquid crystal-based structural color actuators

Structural color actuators are responsive materials constructed from polymers that can be triggered by external stimuli to generate shape and structural color change, either simultaneously or sequentially. Both actuation and structural color can be achieved in the same film using CLCs, CNCs or BPLCs materials. An alternative approach to fabricate structural color actuators is integrating nematic LC based materials with other structural color materials (such as opals). The materials and mechanisms discussed in this review are displayed in Fig. 1.

## Structural color actuators based on chiral photonic liquid crystals

### Structural color actuators based on cholesteric and blue phase liquid crystals

**Fig. 2:**
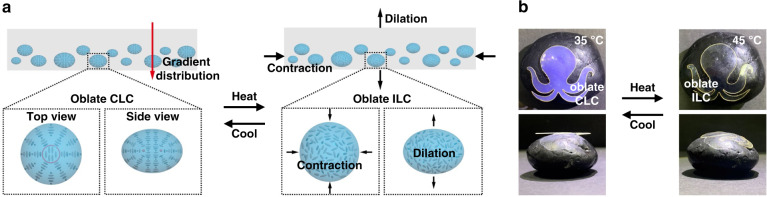
CLC-droplet-dispersed PVA films. **a** Schematic illustration of oblate-CLC-droplet-dispersed PVA films with a gradient distribution of oblate CLC droplets perpendicular to the film plane and thus a gradient thermal expansion perpendicular to the film plane when oblate CLC droplets were heated to the isotropic phase. **b** An “octopus” on a black rock reversibly changed color from blue to colorless and shape from flat to curved for camouflage upon heating and cooling. Reproduced with permission^44^. Copyright 2021, Wiley-VCH

Freestanding structural color actuators can be obtained by dispersing low molar mass CLC droplets in a deformable polymer matrix. For example, dispersing oblate CLC droplets in a polyvinyl alcohol (PVA) polymer matrix allowed both shape transformations (curling) and structural color changes (Fig. 2)^44^. A CLC-droplet gradient distribution through the film depth formed due to gravity (Fig. 2a). Upon heating to the disordered isotropic phase, the ordered oblate CLC droplets transitioned to disordered oblate droplets, causing contraction along the film plane and expansion through the film thickness. Due to the gradient distribution of CLC droplets, the film showed a gradient thermal expansion through the film thickness and a corresponding curling upon heating from 25 to 35 °C with simultaneous droplet color change from red to violet (transitioning from the Sm to the cholesteric phases). This large color change was caused by the pre-transition effect: when nearing the Sm-to-cholesteric phase transition, the pitch approaches infinity; continued heating into the CLC phase caused the decrease of the pitch^40,58^. Further heating to the isotropic phase result in the droplets becoming colorless, having lost the periodic structure required for reflection. A flat ‘’octopus” was demonstrated with reversible curling and color change when heated (Fig. 2b). This fabrication limits shape transformation to a flat initial state and curling at elevated temperature.

**Fig. 3:**
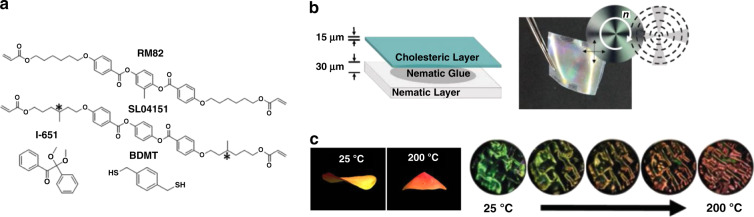
Temperature responsive bilayer structural color actuators based on CLCEs. **a** Molecular structures of the monomers used to prepare the CLCE layer. **b** An illustration of the lamination technique employed to prepare laminated CLCE/NLCE. **c** Topological director profile (left) and photographs (right) of the bilayer film during shape transformation exhibiting concurrent color changes. Reproduced with permission^59^. Copyright 2019, Wiley-VCH

Temperature responsive bilayer structural color actuators have been obtained by laminating a structural colored thin layer of cholesteric LCE (CLCE) onto a nematic LCE (NLCE) actuator^59^. The CLC monomer mixture was photopolymerized within glass cells with planar-aligned alignment layers and detached from the glass plates to obtain a free-standing CLCE film (Fig. 3a). The CLCE film showed thermochromism: upon heating from 25 to 200 °C, the color of the film transitioned from bluish green to red (500–700 nm), caused by the thickness expansion upon heating as the number of pitches is fixed in the polymer network. In the NLCE layer, the alignment of the LC was directed via surface alignment to a predetermined director profile (Fig. 3b), enabling shape transformation from a flat sheet to, for example, a cone upon heating. Concurrent color tuning and shape transformation from 2D to 3D were achieved in the bilayer actuator (Fig. 3c). As the shape change was related to the director profile, the original shape was limited to being flat and the variety of the 3D shapes was determined by the director profiles that can be achieved via this alignment technique.

**Fig. 4:**
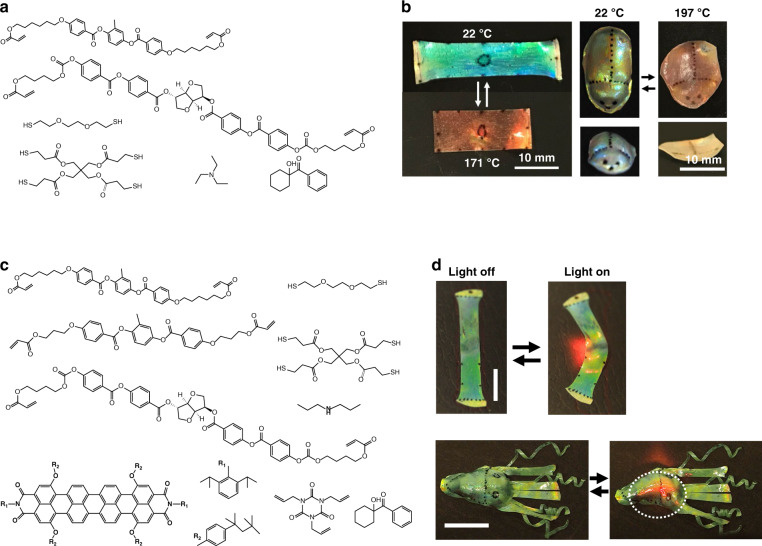
Temperature responsive single layer structural color actuators based on CLCEs. **a** Molecular structures of the monomers used to prepare the CLCE film using a two-stage polymerization. **b** Photographs of a flat CLCEs (left) and a beetle shaped CLCEs (right) at different temperatures. Reproduced with permission^60^. Copyright 2021, Wiley-VCH. **c** Molecular structures of the monomers used to prepare the near-infrared light fueled CLCE film. **d** Photographs of a flat CLCE (top) and a cuttlefish shaped CLCE (bottom) when locally exposed to 780 nm NIR light (scale bars = 10 mm). Reproduced with permission^65^. Copyright 2022, American Chemical Society

Recently, a 4D chiral photonic actuator (with the fourth dimension referring to properties that change in time) was reported using two-stage thiol-acrylate Michael addition and photopolymerization reactions for developing CLCE films with both structural color and shape changes in a single film (Fig. 4a, b)^60^. After the first stage thiol-acrylate Michael addition reaction in a glass cell, the partially crosslinked CLCE film was detached from the glass plates and uniaxially stretched to deform the helical structure as the LC mesogens align along the stretching direction^61–64^. The crosslinking of the film was then completed in the stretched state by photopolymerizing using UV light. The deformed helix resulted in the film reflecting both left- and right-handed circularly polarized light, so-called hyper-reflectivity, which is distinct from classic, non-deformed CLCs where only light with polarization matching the handedness of the CLC helix is reflected. The shape, structural color, and hyper-reflectivity of the CLCE film all underwent reversible changes with temperature. Upon heating from 22 to 171 °C, the CLCE film showed uniaxial-like actuation, with the length (the stretching direction) decreasing by 40% and width (perpendicular to the stretching direction) increasing by 30% due to the thermotropic decrease in order, which is different from the behavior of classic unstretched crosslinked cholesteric films which show both length and width contraction upon heating. Meanwhile, the structural color redshifted from green to red (500 to 680 nm) due to the thickness expansion (Fig. 4b, left). Not limited to being initially flat, objects with 3D shapes were fabricated by molding the partially crosslinked CLCE film before fully crosslinking to fix the shape, which also showed reversible shape change (3D to 2D), structural color shift (green to red) and hyper-reflectivity tuning (Fig. 4b, right). By incorporating a photothermal dye (Fig. 4c), pigmented structural color actuators were prepared where the structural color and shape changes were locally controlled with near-infrared (NIR) light (Fig. 4d)^65^.

**Fig. 5:**
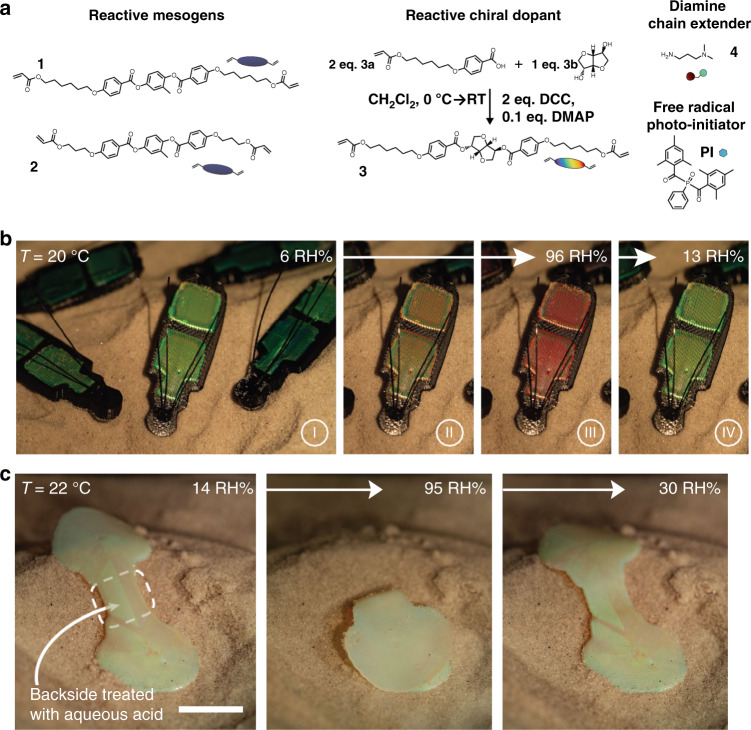
3D printed humidity responsive structural color actuators based on CLCEs. **a** Molecular structures of the components used for synthesizing the cholesteric liquid crystal (CLC) oligomer ink. **b** Photographs of a 3D-printed water-responsive beetles at increasing, and then decreasing, relative humidity. **c** Photographs of the scallop-shaped structural color actuator with increasing, and then decreasing, relative humidity (14–95–30 RH%, scale bar = 5 mm). Reproduced with permission^66^. Copyright 2022, Wiley-VCH

By 3D printing a humidity-sensitive cholesteric liquid crystal oligomer ink (Fig. 5a), a structurally colored actuator was designed^66^. With the relative humidity increasing, the material absorbed water and swelled, causing a redshift in reflected color from green to red (Fig. 5b). A scallop-shaped structural color actuator was also printed and a hinge area between the two complementary shells was selectively treated with acid on the outer side to create asymmetric swelling property in water, allowing reversible “opening” and “closing” when exposed to humid and dry air (Fig. 5c). In this case, the structural color actuator didn’t show color change while actuating.

**Fig. 6:**
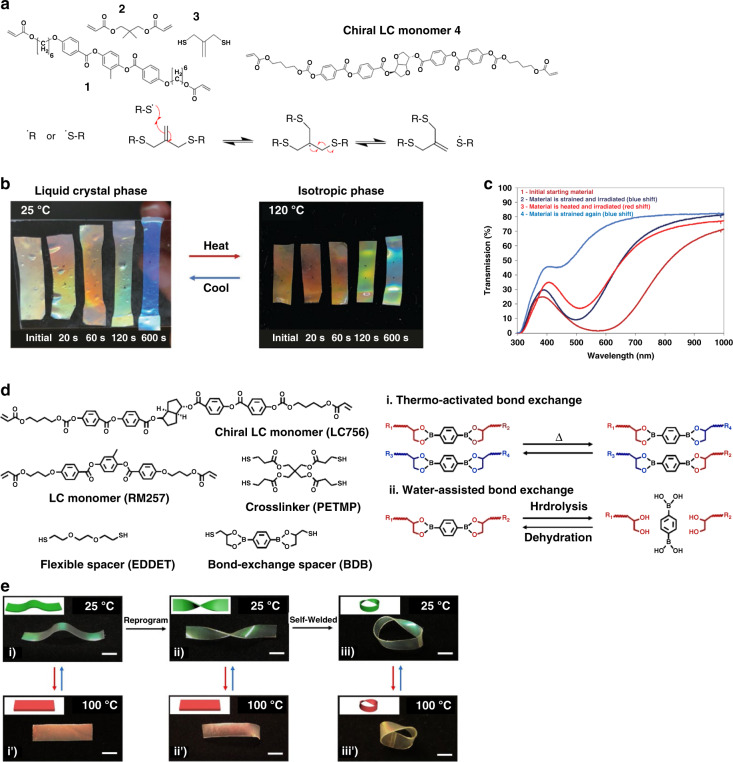
Temperature responsive structural color actuators based on CLCEs with dynamic covalent bonds. **a** Molecular structures of the monomers used to prepare the CLCEs (top). The allyl dithiol (3) gives the CLCE the capability of undergoing reversible AFT bond exchange reactions via the AFT exchange mechanism (bottom): a radical from the photoinitiator or a thiyl radical can induce a bond exchange. **b** Photographs of CLCE films programmed at 25 °C, strained to 100%, exposed with light at 320–390 nm with an intensity of 70 mW cm^−2^ for varying amounts of time (20, 60, 120, 600 s), measured at 25 °C and 120 °C. **c** Transmittance spectra of the CLCE film measured during the programming, cycling between red and blue shifts. Reproduced with permission^67^. Copyright 2020, Wiley-VCH. **d** Molecular structures of the monomers used to prepare the CLCEs (left) and schematic mechanism (right) of B-O bond exchanging in the boronic ester: i) thermo-activated B-O bond exchange; ii) water-assisted B-O bond exchange. **e** Demonstration of reprogrammable and thermo-actuation properties in a single CLCE film (scale bars = 5 mm). Reproduced with permission^69^. Copyright 2022, Wiley-VCH

To achieve reconfigurability, dynamic covalent networks have been incorporated into structural color actuators. By incorporating allyl dithiol, an addition fragmentation chain transfer (AFT) capable species, into the backbone of the CLCE network (Fig. 6a), the shape and structural color of the CLCE were reconfigured to new colors and shapes through bond exchange when activated with UV light^67^. This CLCE displayed both color and shape changes when stretched which were fixed by exposing to UV light, and the degrees of reconfiguration were adjusted by tuning the UV exposure time (Fig. 6b). As the exchange reaction only occurred during radical generation while the UV light was on, spatiotemporal control over both shape and color was demonstrated using a photomask with a striped mask of varying line spacings to achieve patterns in the CLCE: the exposed areas were reprogrammed in a strained state with blue-shifted color while the unexposed areas remained in unstrained states with a red color. The cyclability of the programming was also demonstrated (Fig. 6c): the initial red film was strained to 100% and irradiated with UV for 180 s to program a blue shift. The film was then heated to the isotropic phase at 120 °C and exposed to UV for 10 s to return the film to a red color, after which the film was strained again to 100% to program the blue shift, marked as the second cycle. The transmission decreased after the second cycle due to a loss of alignment. In addition, the cyclability of the programming was limited by the amount of photo-initiator in the material.

In other work, disulfide (S-S) bonds were introduced in CLCE structural color actuators via a two-stage reaction, where LC oligomers with thiol terminal groups were synthesized via the thiol-acrylate Michael addition reaction in the first stage, followed by coupling the thiols to form dynamic S-S bonds in the second stage^68^. This CLCE film exhibited a redshift of the reflection color upon heating due to the thickness expansion. Self-healing was achieved via the dynamic exchange reaction between the S-S bonds under UV light irradiation. Patterns were programmed in the CLCE film with UV light using a photomask to activate the exchange reaction between the S-S bonds in selective areas.

In another example, dynamic covalent boronic ester bonds were introduced into the main-chain CLCE polymer network by using a dithiol-functionalized boronic ester as part of the chain extender of the thiol-acrylate Michael addition (Fig. 6d, left) to achieve self-healable and re-programmable films^69^. The CLCE film obtained showed both reversible shape and structural color changes. The B-O bond in the boronic ester went through an exchange reaction upon thermal activation (Fig. 6d, right), enabling self-healing and reprogramming of the 3D shape (Fig. 6e). For the CLCEs with high molar ratio of dynamic B-O bonds, exchange can also be activated via hydrolysis/dehydration with water, demonstrating good self-healing properties by welding of two CLCE segments at room temperature.

**Fig. 7:**
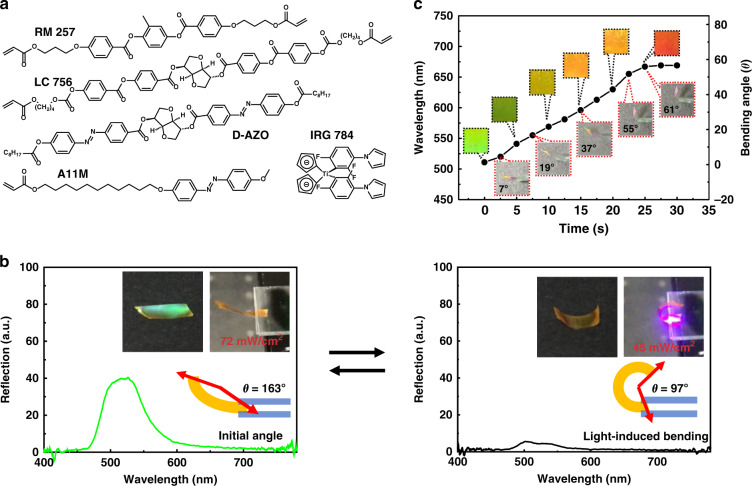
Light responsive structural color actuators based on azobenzene-containing CLC networks. **a** Molecular structures of the monomers used to prepare the CLC networks. **b** Actuation at 25 °C (below T_g_): reflection spectra and photographs of the CLC film with 405 nm light irradiation (right), and after 532 nm light irradiation (left). **c** Actuation at 50 °C (above T_g_): the CLC film exhibited a redshift of the structural color and bending upon 405 nm light irradiation at 50 °C. Reproduced with permission^70^. Copyright 2020, The Royal Society of Chemistry

A light responsive structural color actuator displaying bending and color changes when exposed to 405 nm light was developed using a CLC network containing 57 mol% azobenzene mono-acrylate monomer and 1% azobenzene chiral dopant (Fig. 7a)^70^. When below the glass transition temperature (T_g_), the exposed CLC film bent towards the light source and remained bent after halting the irradiation due to the photoisomerization of the azobenzene side-chain groups. This isomerization also caused the destruction of the planar cholesteric structure, and reflectance decreased from 40% to 10% with the central wavelength remaining at 511 nm, resulting in the film’s perceived color changing from reflective green to yellow from the absorption of the azobenzene (Fig. 7b). When exposed to 532 nm green light, the film returned to its initial state with the green cholesteric reflection restored. When exposed to 405 nm light above T_g_ (50 °C), the azobenzene groups in the chiral dopant underwent photoisomerization, decreasing the effective HTP of the chiral dopant and leading to the expansion of the pitch and redshift of the reflection wavelength from 485 to 670 nm, which was reversible when exposed to 532 nm light (Fig. 7c). Due to the fabrication technique, the original shape of the film was limited to being flat with a slight pre-bend, and the actuation mode was limited to bending.

**Fig. 8:**
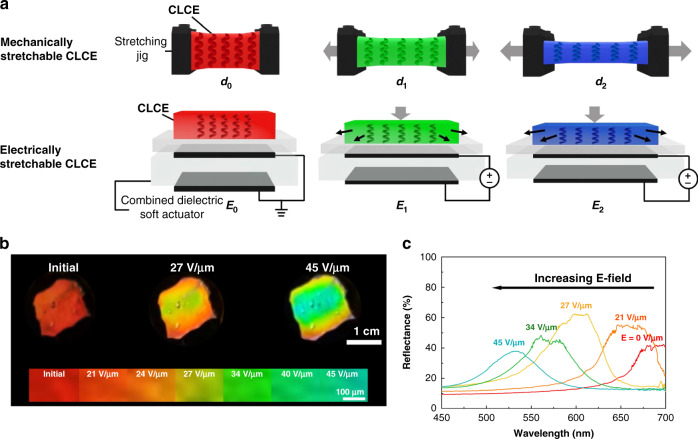
Electrically stretched structural color actuators based on CLCEs. **a** Schematic illustrations showing the reflection wavelength tuning mechanisms for mechanically (top) and electrically stretchable (bottom) CLCEs. **b** Photographs and **c** reflection spectra showing the reflective color change in electrically stretched CLCEs as a function of the applied electric field. Reproduced with permission^71^. Copyright 2021, De Gruyter

Electrically stretchable CLCEs were created using a hybrid structure comprising of elastomeric mesogenic CLCEs on a dielectric soft actuator (Fig. 8a). The actuator consisted of a dielectric elastomer with supporting polydimethylsiloxane (PDMS) sandwiched between two compliant electrodes. When a potential difference was applied between the electrodes, the actuator was compressed in the thickness and stretched in the film plane. This stretching in the dielectric soft actuator induced stretching of the photonic CLCE layer. The CLCEs prepared via a two-stage thiol-acrylate reaction showed blue color shift in reflection upon mechanical stretching due to thickness compression. Reflection band wavelength changes up to 171 nm (from 695 to 524 nm) were observed with an applied electric field strength of 45 V μm^−1^ (Fig. 8b, c)^71^.

**Fig. 9:**
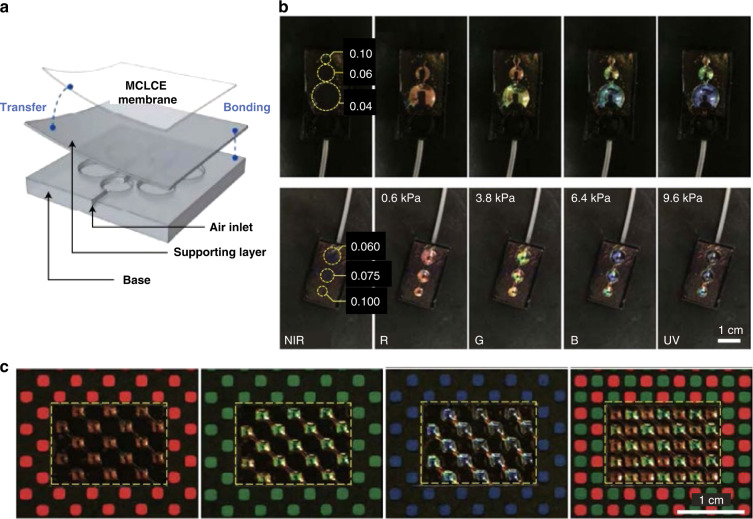
Pixelated camouflage in an inflating CLCE. **a** Schematic representation of the pixelated structural coloration platform, consisting of a base with air channels, a supporting layer, and a main-chain CLCE membrane which was reversibly pneumatically actuated with pressure (p). **b** Top: photos showing simultaneous displays of R, G and B coloration at the same pressure by varying the aspect ratios, t/w = 0.04, 0.06 and 0.10. Bottom: photos showing the reflection spectral shift from NIR to visible and UV. **c** Demonstration of camouflage to match a background with periodic color patterns. Reproduced with permission^72^. Copyright 2021, Nature Publishing Group

Pixelated, inflating CLCEs with broadband spectral shifts were achieved for camouflage purposes^72^. The color unit was a bilayer consisting of a thin layer CLCE film (<15 μm) and a PDMS supporting layer (300–500 μm thick), which was sealed with a PDMS base with embedded air channels for inflation (Fig. 9a). By varying the aspect ratio of the color unit, a variety of spectral shifts in individual pixels were achieved using a shared air channel (Fig. 9b). When the applied pressure was increased to 9.6 kPa, the reflection wavelength shifted from NIR to ultraviolet (UV). Multiple, independent air channels for multiplexed coloration were also demonstrated to match a background for camouflage (Fig. 9c).

**Fig. 10:**
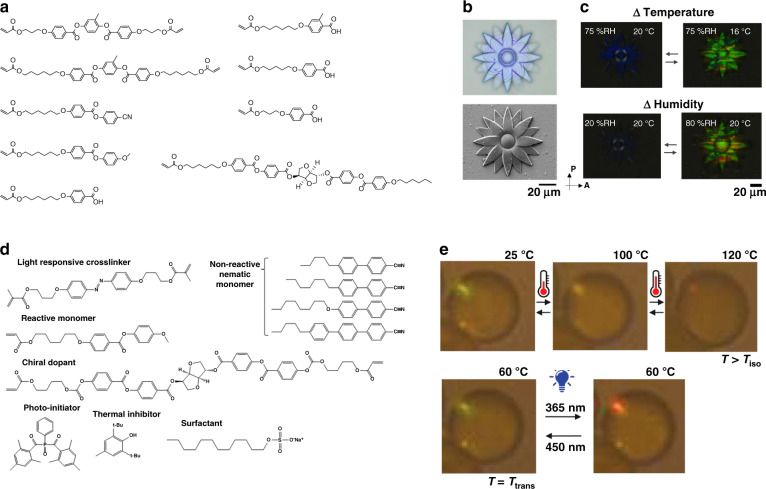
Structural color micro-actuators based on CLCs. **a** Molecular structures of the monomers used to prepare the photonic 3D micro-actuators using direct laser writing. **b** Optical microscopy (top) and scanning electron microscopy (SEM) images (bottom) of a flower-shaped microstructure before base treatment. **c** Crossed polarized micrographs of the flower showing color changes with direct (humidity) and indirect (temperature) triggers. Reproduced with permission^73^. Copyright 2020, American Chemical Society. **d** Molecular structures of the monomers used to prepare the micrometer-sized CLC particles. **e** Temperature (top) and light (bottom) responses of CLC particles exhibiting spot-like structural colored domains. Reproduced with permission^74^. Copyright 2020, Wiley-VCH

The examples mentioned so far in this section are on the centimeter scale. However, structural color actuators on much smaller length scales using CLCs can also be prepared using different techniques. Photonic microactuators (3 to 7 μm), such as pillars, flowers, and butterflies, have been generated using two-photon polymerization direct laser writing of photoresists based on CLC mixtures (Fig. 10a, b); these structures displayed structural color and shape changes when triggered by humidity or temperature (Fig. 10c)^73^. Carboxylic acid mesogens introduced into the CLC network enabled formation of hydrogen bonds, which were then cleaved via base treatment to create a hygroscopic network. When exposed to humidity, the network absorbed water, resulting in a 42% expansion in the film thickness and a corresponding pitch increase of the CLC network, leading to a redshift of the reflection band and a color change from blue to green. Heating removed water from the network, resulting in a decrease in thickness and reflective color blueshift. Temperature was also demonstrated to indirectly actuate the microactuators, as temperature can regulate the rate of water evaporation from the CLC network. For example, when the humidity was kept constant, decreasing the temperature caused a color shift from blue to green.

Dual light and temperature responsive micrometer-sized CLC particles with an average diameter of 7 ± 5 µm were synthesized by suspension polymerization of a reactive CLC monomer mixture containing a light responsive azobenzene dye (Fig. 10d)^74^. The particles exhibited reflective color domains which redshifted upon heating from 25 to 100 °C due to the elongation of the helical structure caused by the liquid-crystalline disorder, and the reflectance disappeared when heated to 120 °C (above the isotropic transition temperature) (Fig. 10e, top). Asymmetric deformations were observed with temperature change, caused by the liquid crystalline disorder. When exposed to 365 nm UV light, the particles exhibited a redshift accompanied by asymmetric deformations (Fig. 10e, bottom), which was reversible upon exposure to 450 nm or to white light. The light responsive color change and deformation were associated with the *trans*-*cis* isomerization of the azobenzene, which caused disorder in the CLC network, resulting in an elongation in the helical direction. The micro-sized structural color actuators could be attractive in application such as optofluidics, optical sensors, and microrobots, and can be produced using CLC mixtures that are quite similar to those forming macro-scale devices except that higher crosslink densities are generally required in the microscopic samples to maintain structure.

**Fig. 11:**
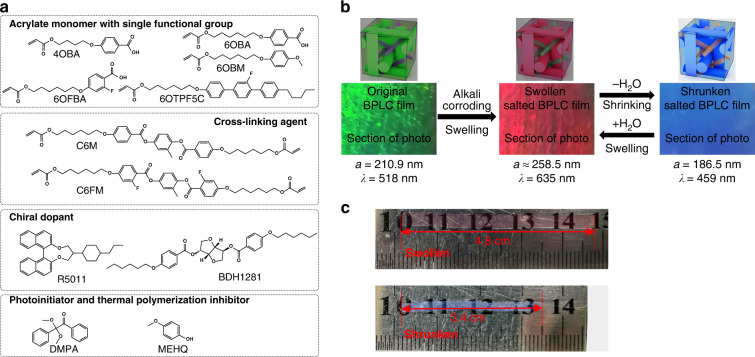
Humidity responsive structural color actuators based on BPLCs. **a** Molecular structures of the monomers used to prepare the BPLC film. **b** Schematic of the mechanism of the responsive behavior of the BPLC film. The original BPLC film exhibited a green color corresponding to a medium lattice size (210.9 nm), which was then corroded by alkali and swelled by water. After swelling, the film turned a red color corresponding to a larger lattice spacing (258.5 nm); upon evaporation of the water, the film shrunk and appeared blue, corresponding to a smaller lattice spacing (186.5 nm). **c** A strip of a BPLC film in the shrunken and swollen states. Reproduced with permission^53^. Copyright 2020, Wiley-VCH

A freestanding BPLC film containing hydrogen bonds was prepared by polymerizing the BPLCs into a network to fix the 3D nanostructures (Fig. 11a)^53^. Upon base treatment, the BPLC film showed reversible humidity-responsive behavior by manipulating the lattice parameters of the nanostructures (Fig. 11b). Upon increasing the relative humidity (RH) from 10% to 80%, the blue film with a center wavelength of 459 nm swelled, causing lattice scale increases and a color change to red with a center wavelength of 635 nm; the film strip expanded from 3.4 cm to 4.8 cm after being completely swollen in water, with an expansion factor of about 40% (Fig. 11c). The BPLC film was also sensitive to pH due to the carboxylate groups, so it could be used for detection of acid gas/liquid such as SO_2_, Cl_2_, and acid rain. Writing and erasing of the BPLC film were also demonstrated.

The examples based on CLCs and BPLCs discussed in this section are summarized in Table 1. In most cases, the structural color actuators are crosslinked, and show redshifts due to thickness expansion when triggered by temperature, light or humidity. However, blueshifts from contraction of the pitch are possible when the structural color actuators are electrically or pneumatically stretched. In structural color actuators involving non-crosslinked CLCs, a blue shift can be observed upon heating, caused by the phase transition from smectic to cholesteric phase rather than a shape change.

**Table 1:** Summary of structural color actuators based on CLCs and BPLCs

Material	Stimuli	Mechanism	Actuation	Structural color	Sensitivity	Ref.
CLC droplets/PVA	Temperature	Sm to Ch transition causes color change, droplets distribution gradient causes curling	Flat to curling	Red to blue	25–37 °C	^44^
CLCE/LCE	Temperature	Thickness expansion causes color change, director profile causes 2D-to-3D shape change	Flat to cone	Green to red (500–700 nm)	25–200 °C	^59^
CLCE	Temperature/NIR light	Thickness expansion causes color change, deformed helix causes uniaxial-like actuation	3D to 2D	Green to red (500–680 nm)	22–170 °C; 22–100 °C	^60,65^
CLCE	Humidity	Thickness expansion causes color change; hinge area for actuation; direct ink writing	Opening to closing	Green to red	14%–95% RH	^66^
CLCE	Temperature	Reprogrammable using AFT reaction	In-plane shape change (2D)	Blue to green (456–517 nm)	25–120 °C	^67^
CLCE	Temperature	Disulfide bond (S−S), pattern, re-programmable	In-plane shape change (2D)			^68^
CLCE	Temperature	Dynamic covalent boronic ester bonds (B-O); thickness expansion causes color change	3D to 2D	Blue to red	25–100 °C	^69^
CLCE	Light	Photoisomerization of azobenzene	Flat to bending	Blue to red (485–670 nm)	405 nm light (45 mW cm^−2^), 532 nm light (75 mW cm^-2^)	^70^
CLCE/soft actuator	Electricity	In-plane expansion of the dielectric soft actuator induces an indirect strain of the CLCE layer	In-plane expansion	Red to green (695–524 nm)	45 V μm^-1^	^71^
CLCE	Pneumatically inflating	Inflation induced stretching causes pitch contraction and blue shift of reflective color	Flat to inflation	NIR to UV	Pressure 9.6–kPa	^72^
CLC network	Humidity and temperature	Hygroscopic CLC network with hydrogen bonds; direct laser writing	Micro-shape (3–7 μm) with 42% height change	Blue to green	75%RH: 16–20 °C; 20 °C: 20%–80% RH	^73^
CLC network	Temperature and light	Thermal induced disorder; trans–cis isomerization of the azobenzene; suspension polymerization	Micro-particles (diameter 7 ± 5 µm)	Green to red, or blue to green	25–120 °C;	^74^
BPLCs	Humidity	Lattice parameters of the nanostructures change when swelling	Swelling	Blue to red	RH: 10%–80%	^53^

### Structural color actuators based on cellulose nanocrystals

**Fig. 12:**
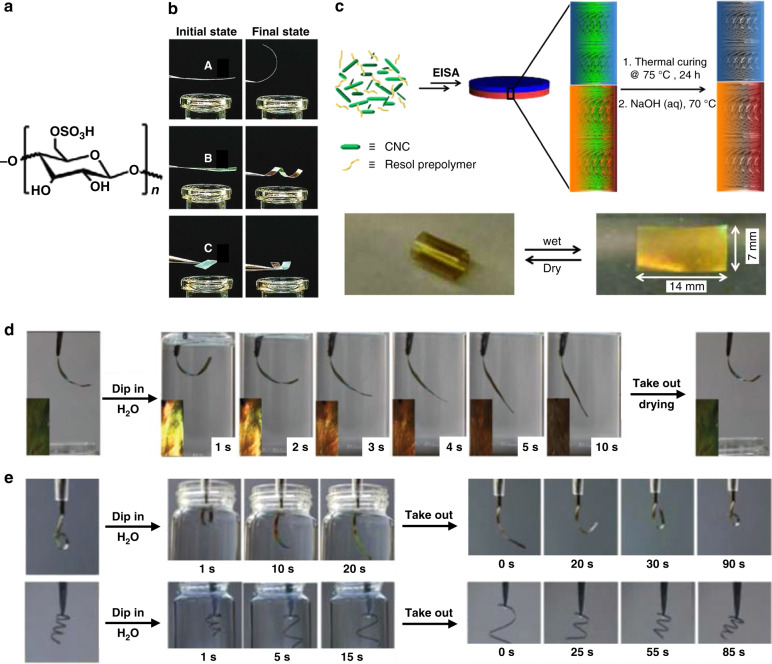
Water/humidity responsive structural color actuators based on CNCs. **a** Chemical structure of CNCs. **b** A sandwiched structure with PA-6 embedded between two CNCs/PEGDA layers showing reversible bending and twisting as well as reflective color change when exposed to a humid environment. Reproduced with permission^75^. Copyright 2016, Royal Society of Chemistry. **c** A CNC/ PF resin composite bilayer with different helical pitch showing curling and color change when exposed to water. Reproduced with permission^48^. Copyright 2014, Wiley-VCH. **d** PF resin/GO-containing CNCs films showing reversible unbending and color change when dipped in water and drying. **e** PF resin/GO-containing CNCs film with predetermined shapes treated with formaldehyde at selective regions and their responses toward a wetting–drying cycle. Reproduced with permission^47^. Copyright 2019, Royal Society of Chemistry

Photonic films with water/humidity-responsive actuation with concurrent optical responsivity can be realized utilizing the asymmetric expansion and contraction between two layers using CNCs (Fig. 12a) deployed either as a material retained in the final films, or as a template which is removed to create mesoporous films with cholesteric order. For example, reversible bending and twisting as well as reflective color change when exposed to humid environments were achieved via asymmetric expansion/shrinkage, realized by embedding a uniaxially oriented polyamide-6 (PA-6) layer between two CNCs/polyethylene glycol diacrylate (PEGDA) layers with left-handed chiral nematic photonic structures (Fig. 12b)^75^. The PA6 layer functioned as a half-wave retarder; thus, the reflectance intensity of the nanocomposite film was able to exceed 50% (hyper-reflection). The hydrophilic nature of CNCs/PEGDA layers allowed the film to swell when exposed to water vapor. The side of the film nearest the moisture source swelled, increasing the helical pitch, while the opposite side of the film remained unchanged, leading to the film bending away from the moisture source and a reflected color change on the stimulated side, which was reversible when the film was removed from the moisture source. By controlling the director orientation in the film by cutting the actuator from a larger sheet at an angle, the deformation of the film could be selected between twisting or bending. In another work, polyethylene glycol dimethacrylate (PEGDMA) was embedded in CNCs to prepare a composite film which showed bending and angle dependent color change when exposed to humidity, due to the asymmetric expansion between the exposed and non-exposed sides^76^. In these examples, the initial shapes of the film were always flat.

In another example with a curled initial shape, bilayer chiral nematic phenol-formaldehyde (PF) resin films were fabricated layer-by-layer using CNCs as a template with helical pitch varying for each subsequent layer (Fig. 12c, top)^48^. By treating the films with NaOH, most of the CNCs templates were removed to create mesoporous resin films with chiral nematic order and double reflection peaks. The films were curled in the initial dry state due to the differential shrinkages between the two layers after the removal of the CNCs templates, where the layer with the larger pitch shrunk more than the layer with the shorter pitch. The high porosity of the resin films after removal of the CNCs endowed the films with good hygroscopicity in polar solvents. The layer with a longer helical pitch had larger pores, and as a result swelled more than the one with a shorter helical pitch. This difference in swelling gave rise to different degrees of expansion and contraction and actuation between uncurling and curling upon wetting and drying: the film was highly curled in the dry state, and upon swelling in water uncurled into a flat film (Fig. 12c, bottom), with a simultaneous redshift of the reflected color (from 390 to 685 nm, and 560 to 970 nm, respectively). The reflected color change could be used for monitoring the degree of actuation.

In the examples discussed above, the initial shape of the film is not controllable. This issue has been overcome in another work where the films were programmed into different initial 3D shapes (Fig. 12d, e). Using CNCs as templates, photonic actuators containing PF resin doped with an asymmetric distribution of graphene oxide (GO) through the film depth were prepared, generating a hydrophilicity difference between the top and bottom surfaces^47^. This hydrophilicity difference resulted in competitive swelling between the top and bottom of the film. The film bent toward the PF resin-rich side in the initial state due to the release of the internal stresses. When dipped in water, the film unbent with a redshift of the reflected color as the PF resin-rich side showed a greater extent of hydration compared to the GO-rich side (Fig. 12d). Selective aldehyde treatment followed by polymerization decreased the flexibility of the selected area due to the increase of crosslink density, allowing forging of the film into desired shapes (the Arabic numeral ‘6’ and a spring) (Fig. 12e), which showed reversible actuation and color changes upon multiple wetting–drying cycles.

**Fig. 13:**
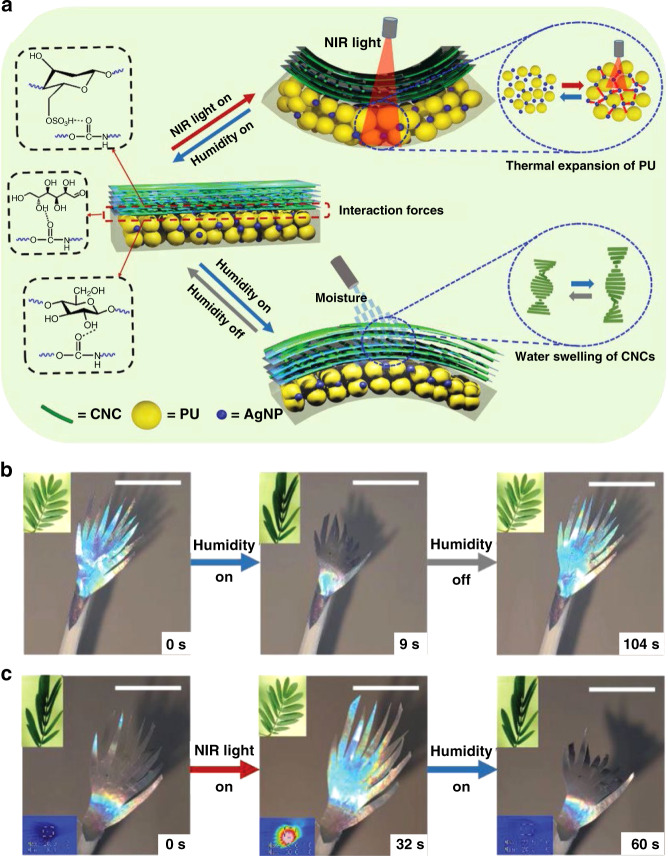
Humidity/NIR light responsive structural color actuators based on CNCs. **a** Schematic mechanism of the CNCs/PU bilayer composite film and its NIR light- and humidity-responsive actuation mechanisms. Photographs of a “mimosa” shaped CNCs/PU bilayer splaying and closing when exposed to (**b**) moisture and (**c**) NIR light. Reproduced with permission^78^. Copyright 2021, Wiley-VCH

By incorporating NIR photothermal agents which convert NIR light into in situ heat via nonradiative relaxation processes (including graphene oxides, gold nanoparticles/nanorods, carbon nanotubes)^77^ into CNCs based structural color actuators, untethered light-driven structural color change and actuation can be realized. NIR light and humidity dual-responsive structural color actuators were developed by laminating self-assembled CNC films to polyurethane (PU) substrates (Fig. 13a)^78^. The CNCs were significantly more hydroscopic compared to the PU due to incorporation of hydroxyl groups. The difference in water absorption and expansion led to the film bending towards the PU with a bending angle of 75° with 299.4 Pa bending force when exposed to humidity, with a color change from blue to red from the pitch expansion upon swelling. Silver nanoparticles were dispersed into the PU to endow the film with additional photothermal response. The PU layer showed a higher coefficient of thermal expansion than the CNCs layer, resulting in the film bending towards the CNCs layer with an angle of 38.4° with a bending force of 199.1 Pa when exposed to NIR light, opposite in direction to the humidity-actuation. A “mimosa” shaped CNCs/PU bilayer was made to demonstrate the NIR light and humidity dual-responsive color change and actuation (Fig. 13b, c). However, only flat, 2D films could be fabricated and more complex 3D initial shapes and versatile actuation beyond bending were not possible.

The examples based on CNCs discussed in this section are summarized in Table 2. Due to the hydroxyl groups in CNCs or hygroscopicity of the polymer matrix, all the CNCs based structural color actuators show humidity/water-responsive swelling, causing thickness increases and pitch expansion and a corresponding redshift of structural color. When deformation results in the viewing angle increasing, the color shift is towards the blue.

**Table 2:** Summary of structural color actuators based on CNCs

Material	Stimuli	Mechanism	Actuation	Structural color	Sensitivity	Response time	Ref.
CNCs/PEGDA and PA-6 sandwiched structure	Humidity	Asymmetric pitch expansion between two layers	Flat to bending/twisting	Green to red (515–735 nm)	80% RH, 25 °C	5 s	^75^
CNCs/PEGDMA	Humidity	Asymmetric expansion between two sides	Flat to bending	Red to green with bending angle increase	Moisture 20 µL s^−1^	4 s	^76^
Bilayer PF resin using CNCs as templates	Water	Asymmetric expansion between two layers	Curling to uncurling	Double reflection 390/560 nm–685/970 nm	Dip in water or water vapor	10 s	^48^
PF resin doped with GO using CNCs as templates	Water	Asymmetric expansion; selective aldehyde treatment to program initial shape	Bending to unbending; 3D shapes to uncurling	600–800 nm	Dip in water	6–180 s	^47^
CNC/PU with AgNPs	Humidity and NIR light	Asymmetric expansion when exposed to humidity and NIR light	Flat to bending	Blue to red	RH 30% - 70%	Humidity: 9 s; NIR light: 16 s	^78^

### Structural color actuators with color and surface topography changes

**Fig. 14:**
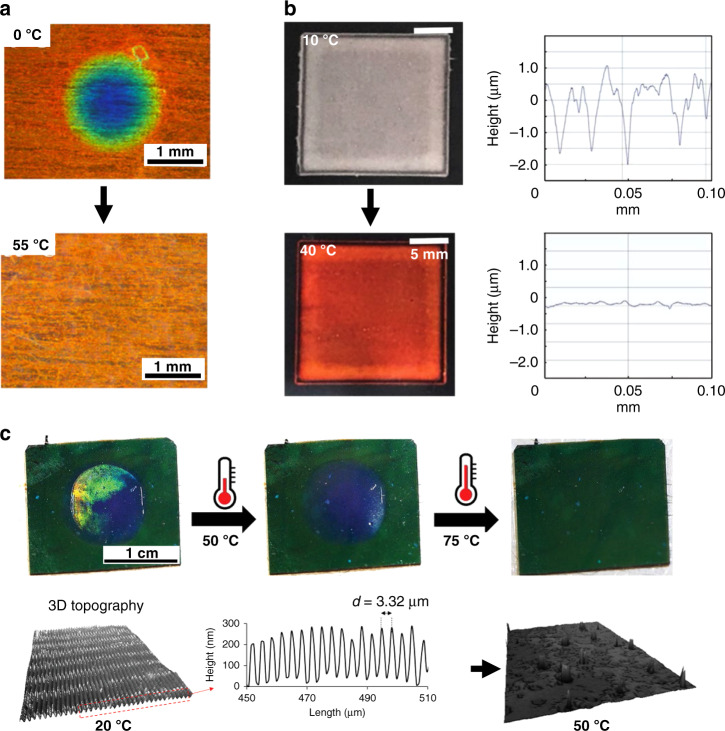
Color and surface topography changes achieved by embossing CLC polymers. **a** Photographs of an embossed CLC polymer upon increasing the temperature from 0 to 55 °C which shows a change in the color from blue to orange. Reproduced with permission^79^. Copyright 2017, American Chemical Society. **b** Photographs (left) and surface topographies (right) of the deformed CLC polymer coating at 10 °C and heated to 40 °C. Reproduced with permission^81^. Copyright 2019, Wiley-VCH. **c** Photographs (top) of the programmed CLC coating showing color and surface topography change (bottom) when heating. Reproduced with permission^82^. Copyright 2020, Wiley-VCH

Embossing a photonic polymer above T_g_ causes thickness/pitch compression, and a blue shift of color, which can be temporarily fixed by cooling below the T_g_. Upon reheating, the polymer irreversibly reverts to its original shape and color. For example, using a semi-interpenetrating network consisting of a CLC polymer network and poly(benzyl acrylate), a blue color shift was achieved, which recovered to red when heated to 55 °C (Fig. 14a)^79^. This has also been done in a crosslinked BPLC^80^. Instead of compressing the polymer film throughout the thickness with a smooth stamp, a surface topography can be embossed using a structured stamp. For example, a rough surface topography was achieved on a red colored shape memory photonic coating^81^. This rough surface topography led to surface scattering and a gray colored coating (Fig. 14b). Upon reheating above the T_g_, the surface recovered to its permanent state, therefore restoring the smooth surface topography and the red reflective color.

Both embossing techniques can be combined in one polymer film to create double photonic effects. This was achieved by dispersing micrometer-sized CLC polymer particles in a shape-memory binder and using a two-step embossing (Fig. 14c). Two different topographies with corresponding structural color changes were created: first compressing the photonic particles at high temperature, causing a blue shift of the structural color, and then embossing a diffractive grating on the surface at an intermediate temperature to create a diffractive topography with a rainbow optical effect^82^. Upon increasing the temperature above the T_g_ of the polymer binder and the CLC polymer particles, the two surface topographies and optical properties sequentially returned to their original states.

**Fig. 15:**
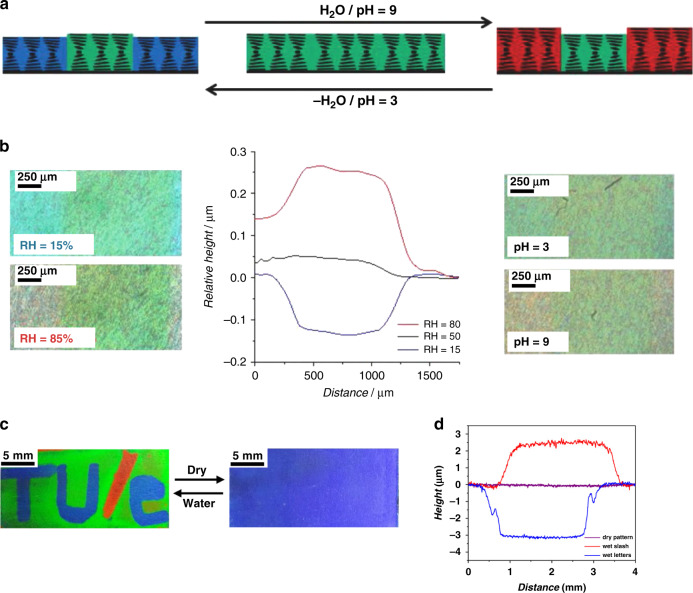
Color and surface topography changes achieved by patterning CLC coatings with local swelling properties. **a** Mechanism schematic of the patterned humidity/pH responsive IPN polymer coating. **b** Left: optical micrographs of a patterned IPN coating at a RH of 15% and 85%. Middle: surface topography of the patterned humidity responsive IPN coating at RH = 15%, 50%, and 80% (*T* = 20 °C). Right: optical micrographs of the patterned IPN polymer film at a pH 3 and 9. Reproduced with permission^83^. Copyright 2015, Wiley-VCH. **c** Photographs of a sponge-written “TU/e” logo that appeared upon exposure to water but remained hidden in the dry state. **d** Height profile of the “TU/e” logo in wet state showed swelling for the slash and shrinkage for the letters with respect to the green background and nearly flat surface in the dry state. Reproduced with permission^85^. Copyright 2018, American Chemical Society

Patterning CLC coatings with local swelling properties or locally printing small molecule inks on top of a uniform CLC coatings can also endow photonic coatings with both color and surface topography changes. An interpenetrating LC-hydrogel polymer network consisting of a CLC network that reflects color and a hydrogel poly(acrylic acid) network that provides a humidity and pH response was reported^83^. The volume change in the hydrogel polymer resulted in a dimensional alteration in the CLC network, leading to a color change (Fig. 15a). A patterned coating with both responsive and static areas was produced, which changed both its surface topography and color when exposed to humidity or pH change (Fig. 15b). In another example, patterned CLC network coatings containing hydrogen bonds were prepared using a photomask and polymerizing different regions at different temperatures, yielding different color and different pitches, and thus a surface topography^84^. Upon exposure to water, the patterns changed their color reversibly, leading to a thickness change at different regions, therefore a topography change.

By printing an aqueous Ca(NO_3_)_2_ solution on top of a CLC network coating containing hydrogen bonds, different patterns were created^85^. The patterns revealed themselves upon exposure to water due to the swelling difference between the printed and un-printed regions (Fig. 15c), with a surface topography change from a flat to a non-flat surface (Fig. 15d). The degree of swelling was controlled by the amount of printed calcium which acted as a crosslinker.

However, a surface topography change is not always related to the structural color change. For example, in a CLCE/LCN semi-interpenetrating network coating, a pillared surface topography was created via polymerization induced diffusion^86^. The reflection intensity of the coating decreased when heated from 30 to 120 °C due the cholesteric-to-isotropic transition, resulting in a weaker red color. The surface topography transitioned from hills to flat to valleys when heating from 23 to 50 °C due to a crosslink density difference-induced thermal expansion difference between the pillars and the surroundings.

An overview of this section can be found in Table 3. Surface topographies and color changes can be introduced by embossing CLC or BPLC networks; the original structure is restored by heating, restoring the original color at the same time. By creating a patterned film with different local swelling properties, surface topographies in a polymer film can also be achieved. When exposed to water/humidity, the patterned film shows distinct swelling with different pitch expansions, leading to surface topographies and different degrees of structural color redshifts.

**Table 3:** Summary of structural color actuators with structural color and surface topography changes

Material	Stimuli	Mechanism	Actuation	Structural color	Sensitivity	Ref.
CLC network/poly(benzyl acrylate)	Temperature	Embossing and shape memory	Height increasing	Blue to red	0–55 °C	^79^
BPLC network	Temperature	Embossing and shape memory	Height increasing	Blue to red	0–40 °C	^80^
CLCE	Temperature	Embossing with rough surface and shape memory	Rough to smooth surface	Reflection intensity decreasing	10–40 °C	^81^
CLC polymer particles/polymer binder	Temperature	Two-step embossing and shape memory	Height increasing, diffractive grating topography to smooth	Rainbow effect vanishing; blue to green	20–50–75 °C	^82^
Interpenetrating LC-hydrogel networks	Humidity/pH	Absorption of water causes pitch change, pattern	Surface topography change	497–650 nm; 563– 730 nm	RH: 6%–85% pH: 3–9	^83^
CLC network with hydrogen bonds	Water	Absorption of water causes pitch change, pattern	Thickness increasing	520– 720 nm	Water	^84^
CLC network	Water	CLC network swelling in water	Flat to non-flat surface	Blue to red	Water or breath	^85^
CLCE/LCN semi-interpenetrating network	Temperature	cholesteric-to-isotropic transition causes reflection intensity decreasing; crosslink density difference-induced thermal expansion difference causes topography change	Pillars to valleys	Reflection intensity decreasing	Color: 30–120 °C; Topography: 24–50 °C	^86^

## Structural color actuators based on liquid crystals with opal/inverse opal structures and other microstructures

Structural color actuators can be achieved by laminating LCNs/LCEs with opal photonic crystals or using opals as templates to create an LCN/LCE with an inverse opal bilayer structure. As discussed earlier, planar-aligned LCNs/LCEs show thermal-driven contraction along the nematic director due to thermotropic disruption of order, while the opal layer shows almost no dimensional changes, resulting in the asymmetric shrinkage/expansion of the bilayer structure and a bending deformation towards the LCE layer. At the same time, the opal or inverse opal structure packs more closely, leading to a decrease in lattice spacing due to the bending, therefore a reflective color blueshift as the reflection wavelength is related to the lattice constant.

**Fig. 16:**
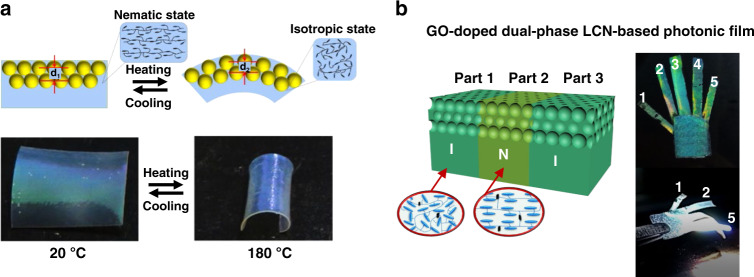
LCN with opal or inverse opal structures. **a** Top: schematic illustration of the actuation and dynamic change of the lattice distance in the SiO_2_ opal/LCN composite films. Bottom: photographs of the reversible actuation and color change of the SiO_2_ opal/LCN composite film induced by temperature variation. Reproduced with permission^87^. Copyright 2016, American Chemical Society. **b** Schematic of the dual phase LCN photonic films (left) and photographs of finger action of a bionic hand based on the LC photonic films (right). Reproduced with permission^91^. Copyright 2018, Wiley-VCH

Structural color actuators with opal structures can be achieved using different nanospheres/nanoparticles. Silica opal spheres were embedded into a LCN network to introduce structural color in nematic LCNs. A bilayer SiO_2_/LCN composite film was reported that showed bending as well as a color change from 535 to 519 nm when heated from 20 to 180 °C (Fig. 16a)^87^. A monolayer of silver nanoparticles (AgNPs) was also deployed on the surface of a LCN as surface arrays to make an actuator with dual deformation and color changes^88^. By varying the LCN alignment between homeotropic and splay (planar alignment on one side of the film and homeotropic on the other), the film can exhibit expansion or bending deformations when heating, each accompanied with color changes. Photonic polystyrene (PS) nanospheres were laminated onto an LCE layer doped with carbon nanotubes as photothermal agents. This PS laminated LCE composite film showed bending and a blueshift of color (21 nm) under infrared irradiation^89^.

To prepare an LCN film with inverse opal structure, silica was first embedded in the LCN and then removed using dilute hydrofluoric acid, yielding a microporous structure with closely packed vacant spheres. Both bilayer or single layer LCNs can be prepared, depending on whether the opal template is distributed throughout or only present in part of the LCN. For example, a bilayer consisting of two LCN layers, one with no inverse opal structure and one with inverse opal structure was prepared. The film showed bending because of the asymmetric contraction between the layer with and without inverse opal structure when heating, causing more dense packing of the inverse opal pores and a blueshift of the reflective color (less than 20 nm)^90^. By doping the LCN with the photothermal agent GO, the bilayer LCN film with partial inverse opal structure close to the film surface could also be triggered with light, bending 60° and undergoing a reflective wavelength shift of 15 nm (from 559 to 544 nm)^91^. Selective actuation was achieved by photopatterning the LCN locally in nematic (N) and isotropic (I) phases, allowing for different local bending behaviors (Fig. 16b). However, the initial shape was limited to being flat. The color change in the bilayer LCN with opal/inverse opal structure is restricted, normally less than 30 nm in wavelength, as the change of lattice spacing caused by bending is limited.

A single layer LCN film with inverse opal structure was fabricated with the opal template distributed throughout the thickness of the LCN. Using silica as template, a polydomain LCN film containing azobenzene with inverse opal structure was prepared^92^. When exposed to 365 nm UV light, the azobenzene photoisomerized from *trans* to *cis*, causing disorder in the LC and contraction of the film surface, and the LCN film bent towards the light due to the contraction gradient through the thickness. This disorder disturbed the periodic holes in the inverse opal film, causing the reflection intensity (at 677 nm) to decrease from 90% to 20%. This disorder could also be triggered by temperature: when heated to 90 °C, the film contracted in the plane and the reflection intensity almost disappeared from 90% to less than 10%. This difference in actuation and decrease in reflection was due to the UV light creating a gradient of disorder through the film depth while heating induced more uniform disorder. However, the intensity of the refection peak only partially recovered (to 70%) when exposed to 530 nm light or cooling. When inverse opal LCN films were attached to glass plates, bending deformation was not possible and the films instead underwent expansion through the thickness when exposed to stimuli such as temperature or electric fields, leading to increasing lattice distance of the pores and a redshift of structural color^93,94^.

**Fig. 17:**
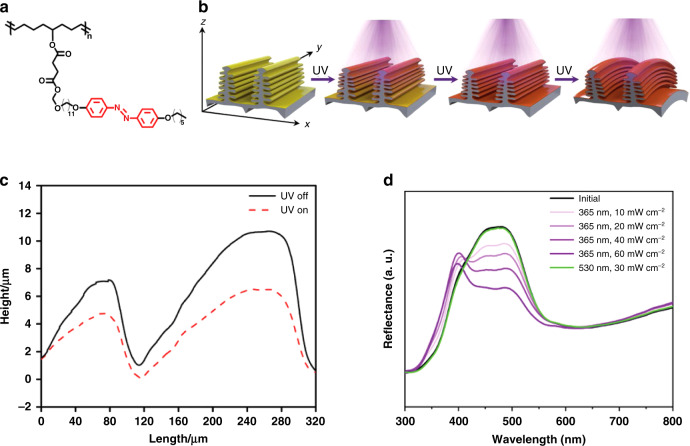
LCs on hierarchical microstructures. **a** Chemical structure of the photo-responsive LCP containing azobenzene mesogens. **b** Schematic showing the deformation of the bilayer LLCP-MBW. With the increase of UV intensity, the penetration of incident light gradually increased into the lamellas of the ridges, causing the deformation of microstructures and the gradual increase of the 397 nm reflection peak. **c** Surface profiles of the LLCP-MBW show the reversible deformation of the scales on the LLCP-MBW upon UV (365 nm, 10 mW cm^−2^) and visible light (white light from the microscope) irradiations. **d** The changes of reflectance spectra of LLCP-MBW after 365 nm UV irradiation with different intensities for 10 s and subsequent 530 nm visible light irradiation for 10 s. Reproduced with permission^96^. Copyright 2019, Wiley-VCH

By creating microarrays on an LCN surface, structural color was introduced in non-photonic LCN films. An azobenzene-doped LCN film topped by 1 μm period micropillars was reported using a replica molding technique; the diameter of the pillars increased 18% and the reflection intensity decreased from 34.3% to 18.9% when exposed to UV light, caused by the *trans* to *cis* photoisomerization induced disorder^95^. A photo-responsive composite was achieved by electrospinning an azobenzene-containing LC polymer (LCP, Fig. 17a) mixture onto the surface of a Morpho butterfly wing (MBW) template^96^. Upon UV light irradiation, the perpendicularly oriented azobenzene underwent *trans-cis* isomerization, deforming the LCP layer with a thickness decrease and in-plane expansion, resulting in the MBW bending away from the light (Fig. 17b). This deformation caused the spacing of the microstructures to change (Fig. 17c), resulting in a change of the reflection properties: the reflection peak at 470 nm decreased 40% and a new peak at 397 nm appeared; the intensity of this new reflection peak increased with increasing UV intensity from 10 mW cm^−2^ to 60 mW cm^−2^ (Fig. 17d).

An overview of this section can be found in Table 4. The structural color change of LCs with opal/inverse opal structures is limited, usually less than 35 nm, and actuation is limited to in-plane contraction or bending. In the few examples of structural color actuators produced from integrating other microstructures onto/into the material system, such as micro-pillars or butterfly wings, the structural color only involves intensity changes or restricted color shifts.

**Table 4:** Summary of structural color actuators based on nematic LCs with opal/inverse opal structures and other microstructures

Material	Stimuli	Mechanism	Actuation	Structural color	Sensitivity	Other	Ref.
LCE/silica opals	Temperature	Asymmetric contraction between LCE and silica	Flat to bending	535–519 nm	20–180 °C	–	^87^
LCE/AgNP particles	Temperature	Thermal induced disorder of the LCE causes deformation; distance increasing between particles caused by deformation induces color change	Expansion (homeotropic alignment) or bending (splay alignment)	Contraction: 531–507 nm; bending: 531–498 nm)	30–130 °C	–	^88^
LCN/inverse opal structure (bilayer)	Temperature	Asymmetric contraction between LCN and inverse opal layer; more closely pores of the inverse opal when bending induces color change	Flat to bending	527–511 nm	25–100 °C	–	^90^
GO doped LCN/inverse opal structure (bilayer)	Light	Asymmetric contraction between LCN and inverse opal layer; more closely pores of the inverse opal caused by bending induces color change	Flat to bending, selective bending via photopatterning	559–544 nm	Visible light 100 mW cm^−2^ (84.6 °C)	Response time 3 s; self-oscillation via self-shadowing	^91^
Azobenzene containing LCN with inverse opal structure (single layer)	Temperature and light	Thermal/photo-induced disorder of the LCN causes the order of the periodic structure of the holes decrease	Bending with UV light on, contraction with heating	Reflection intensity decreasing: 90%–20% (UV light), 90%–0% (heat)	UV light (365 nm, 50 mW cm^-2^); temperature 30 - 90 °C	–	^92^
LCN with microarray	Light	Trans-cis photoisomerization of the azobenzene causes the shape change of the LCN	Diameter of the pillars increases by 18%	Reflection intensity decreasing: 34.3%–18.9%	365 nm, 20 mW cm^−2^	Response time 15 min	^95^
LCP on Morpho Butterfly Microstructures	Light	Trans-cis photoisomerization of the azobenzene causes bending and spacing change of the microstructures	Tilted angle of the scales decreases with a height drop of 4 µm	A new reflection at 370 nm and a 35% decrease at 470 nm	365 nm, 60 mW cm^−2^;530 nm, 30 mW cm^−2^,	Response time 10 s	^96^

## Conclusion and perspective

There are many examples of LC based structural color actuators capable of changing color while actuating. The triggers for these structural color actuators include temperature, water/humidity, light, electricity and stretching; water/humidity and temperature responses dominate the current literature.

Chiral photonic LCs are the most common LC materials used to make structural color actuators, with simplified production due to their self-assembly and potential of large color shifts (up to 200 nm). The CLC based structural color actuators can even be programmed into different initial 3D shapes, enabling diverse actuation modes^60,65,69^. In addition, using dynamic covalent chemistry, reconfigurability has been achieved^67–69^. CNCs, in contrast, are generally brittle and must be integrated into other polymers or be used as templates to create porous structures to allow flexibility. However, CNCs are biobased materials and their prevalence in nature bodes well for introduction in materials that will interact with living matter. Opal/inverse opal structures generally require bilayers or removal of templates and increased production complexity with somewhat limited spectral shifts with color shifts usually below 35 nm, and actuation is limited to bending or in-plane contraction. Finally, there are only limited examples of structural color actuators produced from integrating other microstructures onto/into the material system, such as micro-pillars or butterfly wings: in these devices there is only a change in reflection intensity, and actual color shifts are quite limited. In summary, while there are several alternatives to CLC actuators, currently they are the apparent best option when considering flexibility in design, actuation magnitude, breadth of triggering stimuli, and potential color shifts.

Production of structural color actuators has been generated primarily from making cells or deposition by bar or spin coating on surfaces. Some new options are becoming available, including direct ink writing for macroscopic actuating objects (called “4D printing”, with the fourth dimension being time)^97^, and two-photon processes for microscopic objects^73,98^. One advantageous feature from all these production techniques is that the liquid crystalline mixtures are not all that different, and so adapting mixtures for different deposition techniques is much simplified.

There are a few examples of using structural color actuators in soft robots for camouflage, an important feature for many animals to survive or signal to each other. For example, the octopus-shaped CLC-droplet-dispersed PVA films (Fig. 2) emulate camouflage as they show color change and curling to emulate a rock^44^. The pixelated camouflage demonstrated in the inflating CLCE (Fig. 9)^72^ goes even further, with a highly desirable broad spectral shift for adaptation to an ever-changing background. In most structural colored actuators to date, the color change results from altering lattice distances and/or refractive indexes of photonic structures, and therefore is coupled with shape change. This is not desirable for achieving on-demand camouflaging, where orthogonal shape and color changes are needed. Solving this problem will enable photonic materials to achieve more complex life-like camouflage while maintaining independent actuation. It is difficult to decouple them within a single actuator, so one must think in terms of bilayers, in which each layer responds to different triggers, for example, one causing bending and one generating color changes. An example has been shown in an LC actuator used to change the orientation of cholesteric liquid crystal layers co-deposited on a substrate^99^: by modifying the cholesteric to be responsive^66^ in this construction will bring truly decoupled structural color actuators. The structural color change in the actuators can also be used for noncontact visual measurement of the movement of soft actuators/robots: for example, the bending angle of the soft actuators can be determined by monitoring the color of the film (Figs. 7c and 12d).

To move towards interacting actuators in soft robotic devices, it is necessary to find approaches to achieve communication/interaction between soft actuators or between a soft actuator and its surrounding. The capability of light regulation, that is, self-sensing in structural color actuators can be used to achieve local interaction. However, the challenge lies in the weak optical signal encountered by the object and one must find a solution to amplify the optical signal or improve the sensitivity of the soft actuator to realize communication/interaction. In reported non-LC based structural color actuators, there is some progress for self-sensing of the environment by changing the structural color, e.g., hydrogel based iRobot monitoring the temperature change in the environment by change of the body color^100^ or the solvent vapor driven walker which changes body color while walking^101^. However, the optical signal is not utilized to trigger further action.

Utilizing interaction between soft actuators and the environment to achieve self-regulation, the ability to sense the environment and react autonomously, is another opportunity. Imagine an artificial soft robot moving on a surface which could modify its skin color and body shape to match the background or change its behavior, such as movement speed^102^ to react to the environmental changes, such as humidity, roughness and reflectivity of the track. To achieve this, designing a structural color actuator assembly able to move itself is essential. Among all the stimuli, light stands out for this scenario, as the interaction between the light utilized to trigger the structural color actuators and the light reflected by the structural color actuators will allow a tremendous design space to achieve interacting soft robots. More efforts will be required to utilize the self-sensing capability of structural color actuators to bring more versatility, and allow interaction with its environment, learning from the inputs it receives and self-regulation of its own actuation^103^.
